# Barrier Film of Etherified Hemicellulose from Single-Step Synthesis

**DOI:** 10.3390/polym12102199

**Published:** 2020-09-25

**Authors:** Hui Shao, Yuelong Zhao, Hui Sun, Biao Yang, Baomin Fan, Huijuan Zhang, Yunxuan Weng

**Affiliations:** 1 College of Chemistry and Materials Engineering, Beijing Technology and Business University, Beijing 100048, China; sh050305@126.com (H.S.); 13552134315@163.com (Y.Z.); fanbaomin@btbu.edu.cn (B.F.); zhanghuijuan@btbu.edu.cn (H.Z.); wyxuan@th.btbu.edu.cn (Y.W.); 2 Beijing Key Laboratory of Quality Evaluation Technology for Hygiene and Safety of Plastics, Beijing Technology and Business University, Beijing 100048, China

**Keywords:** hemicellulose, barrier film, etherification, epoxy chloropropane

## Abstract

Hemicellulose with good biodegradability and low oxygen permeability shows great potential in food packaging. However, its strong hydrophilicity leads to its poor moisture resistance, which hinders its wider application. In this paper, a near-hydrophobic hemicellulose was obtained by using single-step synthesis from poplar powder via etherification modification with epoxy chloropropane. This proposed approach has the advantage of avoiding the destruction of hemicellulose structure by secondary alkali-hydrolysis, which was what usually occurred in traditional etherification procedures. The feasibility of using epoxy chloropropane as an alkylation reagent to etherify hemicellulose was confirmed, and the reaction mechanism was elucidated. Contact angle test, thermogravimetric analysis, oxygen transmittance test, and infrared spectrum analysis showed that the barrier property and thermal stability of etherified hemicellulose films have been significantly improved. At an epoxy chloropropane/wood powder ratio (volume/weight) of 2/3 (mL/g), the epoxy hemicellulose films contained the most epoxy groups and displayed the best performance, i.e., tensile strength of 14.6 MPa, surface contact angle of 71.7° and oxygen transmission coefficient of 1.9 (cm^3^·µm)/(m^2^·d·kPa), showing great promise as barrier film in food-packaging.

## 1. Introduction

In the face of the exhaustion of petrochemical resources and the pressure brought to the environment by the extensive use of petroleum-based packaging materials, development of renewable packaging materials of biomass origin has been a research focus. Utilization of forest biomass in this regard is expected to effectively avoid the low-efficiency use of abundant biomass resources, which reduces the dependence on petrochemical resources, and thus ensures energy security [1,2,3,4,5,6]. Hemicellulose is a kind of natural macromolecular material with plentiful sources. However, because of the complexity and diversity of hemicellulose structure, the research on cellulose and lignin has occupied the main position in the research on forest biomass for a long time [2,5,7,8], and the research on hemicellulose extraction and high-value utilization is relatively lagging behind [9,10]. Through continuous research, the utilization of hemicellulose has become possible [11,12,13]. Hemicellulose film as packaging material is essentially still in the research stage, although preliminarily applications have been found, e.g., as cover film to keep pepper fresh [14].

Hemicellulose is composed of a variety of glycan structures: including xyloglucan, xylan; mannannan and glucomannan; β-(1→3,1→4)-glucan; etc., [15]. The presence of a large number of hydroxyl groups in hemicellulose molecules results in the strong designability of this biopolymer. With the addition of plasticizer [16] and enhancer [1,17,18], the application of hemicellulose film became possible. However, the large number of hydrophilic hydroxyl groups make hemicellulose film susceptible to the absorption of moisture, resulting in poor performance when used in a humid environment [19]. Hemicellulose can be chemically modified by etherification [20,21,22], esterification [23,24,25], grafting [26,27], sulfonylation, and such [28], to improve the moisture resistance of hemicellulose films. For example, the contact angle of the hemicellulose film after the citrate esterification and cross-linking can reach 87.5° [29], and the grafting-modified hemicellulose film has a contact angle as high as 81° [30]. In addition, the water vapor permeability of lauric acid hemicellulose film [14] and borate cross-linked hemicellulose film [31] decreased significantly, and even reached the level of traditional barrier films [32]. Epoxides have received extensive attention in food packaging materials, and preliminarily applications have been found [33,34]. At the same time, attention was paid to the safety of epoxides in food packaging materials by researchers [35]. Epoxides possess an unstable ternary ring containing oxygen, which causes electrons to fall off neighboring carbon atoms; thus, epoxides are reactive to the hydroxyl compound, hemicellulose. The etherification can reduce the solubility of the hemicellulose film, enhance its biodegradability, and improve its film-forming performance [36].

Hydroxyl groups on hemicellulose can act as electrophiles to react with epoxy chloropropane. Traditional etherification requires the extracted hemicellulose to be dissolved in lye before reacting with epoxy chloropropane. Secondary alkali-hydrolysis will result in the destruction of hemicellulose structure, a decreased degree of polymerization, and poor mechanical properties [37]. In view of this drawback, this paper reports on the modification of hemicellulose by etherification in the process of hemicellulose extraction from poplar powder residue by one-step method, where the modified product was obtained by direct alcohol precipitation, avoiding the damage to poplar hemicellulose by secondary alkali-hydrolysis. In a subsequent solution casting procedure, sorbitol as a plasticizer of small molecules, hemicellulose macromolecules, and polyvinyl alcohol (PVA) were self-assembled through non-covalent bonds to achieve regular structures at different scales. Hemicellulose-based films with good properties of safety, nontoxicity and resistance were obtained, and their barrier performance, mechanical, and heat-resistant properties were characterized.

## 2. Materials and Methods

### 2.1. Materials and Reagents

Poplar wood powder with particle size between 0.2 mm–0.8 mm was collected from five-year poplar in Hebei province with hemicellulose content of 30.5%. The main components of hemicellulose were 4-*O*-methyl glucuronic acid xylose. Sodium hydroxide, sodium chlorite, hydrochloric acid, sorbitol and epoxy chloropropane (of analytical purity, purchased from Sinopharm Chemical Reagent Co., Ltd., Shanghai, China), glacial acetic acid (Beijing Chemical Plant, Beijing, China), ethanol (95%, *w*/*v*, Tianjin Oko Chemical Reagent Co., Ltd., Tianjin, China) and PVA (with degree of polymerization of 1700 and alcoholysis degree 99%, China Petrochemical Co., Ltd., Beijing, China) were all used as received.

### 2.2. Extraction and Etherification of Poplar Hemicellulose

**Figure 1:**
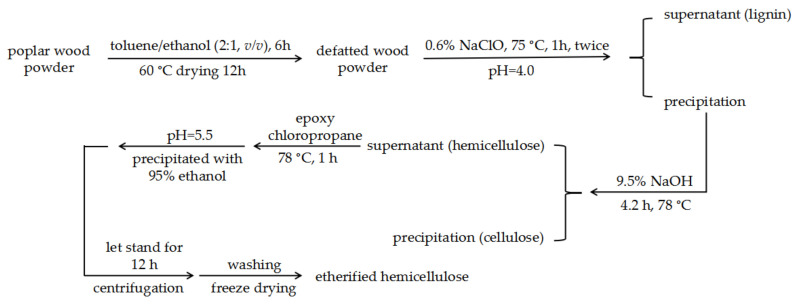
The flow chart of single-step synthesis etherified hemicellulose.

The flow chart of single-step synthesis etherified hemicellulose is shown in Figure 1. Dried poplar powder was Soxhlet-extracted for 6 h using toluene/ethanol (2:1, volume ratio) and then dried for 12 h at 60 °C. In a 75 °C water bath, the solid–liquid ratio 1:20 (*w*/*v*) was maintained, the pH was adjusted to 4.0 with glacial acetic acid, and the defatted wood powder was extracted twice with 0.6% (*w*/*v*) sodium chlorite solution, each time for 1 h. After filtration, the filter residue was cleaned and dried to obtain the lignin powder. Then, 60 g of powder was left in 9.5% (*w*/*v*) NaOH solution for 4.2 h at 78 °C. The solid–liquid ratio was also kept at 1:20 (*w*/*v*).

After alkali extraction and filtration, 0 mL, 5 mL, 10 mL, 20 mL, 40 mL, 60 mL, and 80 mL of epoxy chloropropane was added dropwise into the filtrate at 78 °C for 1 h. The feed ratio of epoxy chloropropane (mL/g) is the ratio of the volume (mL) of epoxy chloropropane to the mass (g) of the delignificated wood powder.

After the pH was adjusted to 5.5 with hydrochloric acid, the filtrate (containing etherified hemicellulose) was precipitated with 95% (*w*/*v*) ethanol (1:3 *v*/*v*). The mixture was left to stand for 12 h before polar hemicellulose was obtained by centrifugation, followed by the drying of the filter residue.

### 2.3. Preparation of Etherified Hemicellulose Films

PVA was pre-dissolved in water at 95 °C in a beaker. Then hemicellulose and sorbitol were added, and the mixture was stirred for 4 h at 75 °C. After ultrasonic treatment for 10 min (59 kHz), the solutions were cast into polystyrene culture dishes (130 mm × 130 mm) and vacuum defoamed for 15 min. The main composition of each film is as follows: 60% poplar hemicellulose (*w*/*w*), 20% PVA (*w*/*w*), and 20% sorbitol (*w*/*w*). The total mass of the above three components was 1.5 g (Table 1). Water was evaporated in an oven at 50 °C and the dried films were left to stand overnight at room temperature prior to all measurements.

### 2.4. Analytical Methods

**Determination of epoxy value:** According to China national standard GB/T 1677-2008, 1 mL of hydrochloric acid was dissolved in 40 mL of acetone to obtain acetone/hydrochloride solution. The sample of about 1 g was put into a conical flask with stopper, and 20 mL of acetone/hydrochloride solution was pipetted. The mixture was shaken and left to stand for 1 h. Three drops of phenolphthalein indicator solution were added, and the solution was titrated with 0.1 mol/L NaOH standard solution from colorless to pink as the endpoint. The blank test was conducted at the same time.

(1) $$\mathrm{The}\;\mathrm{epoxy}\;\mathrm{value}\;(\mathrm{EPV})=\frac{(V_{0}-V)C}{10W}$$

In the above equation, *V* is the volume of standard sodium hydroxide solution consumed by the sample (mL), *V*_0_ is the volume of standard sodium hydroxide solution consumed in blank test (mL), *C* is the equivalent concentration of sodium hydroxide standard solution (mol/L), and *W* is the weight of the sample.

**Infrared spectrum analysis (FT-IR):** The chemical functional groups of hemicellulose were analyzed on a Fourier infrared spectroscopy analyzer (iN10 MAX, Thermo Scientific Co., Ltd., Shanghai, China). The spectra were obtained at a resolution of 4 cm^−1^ with 32 scans in the range from 4000 cm^−1^ to 450 cm^−1^.

**Thermogravimetric analysis (TGA):** Thermal stability tests of the films were carried out on a thermogravimetric analyzer (Q50, TA Instruments Inc. New Castle, PA, USA). The test temperature ranged from 40 °C to 600 °C with the nitrogen flow rate maintained at 100 mL/min and the heating rate of 20 °C/min.

**Tensile test:** According to China national standard GB/T 1040.2-2006, the film sample was cut into a rectangular specimen of 10 mm × 80 mm. The tensile test of the films was performed with a universal material testing machine (CMT6104, MTS Systems Co. Ltd., Wuhan, China). The initial distance between the grips, and the cross-head speed, was kept constant at 80 mm and 5.0 mm/min, respectively. Tensile strength and elongation at break values of the films were averaged over five specimens.

**Oxygen permeability (OP) measurement:** The oxygen barrier properties of the composite films were assessed on a circle sample surface (10 cm in diameter) by use of a VAC-V2 permeability analyzer (OX-TRAN 2/21, Mocon Inc., Minneapolis, MN, USA). The measurements were conducted at 23 °C under 50% relative humidity (RH) condition according to the standard method described in China national standard GB/T 1038−2000. The OP is given in units of [(cm^3^·µm)/(m^2^·d·kPa)]. Each result was averaged over three specimens.

**Contact angle test:** Contact angles were measured on a goniometer equipped with a measuring video system (OCA35, DataPhysics Instruments GmbH, Beijing, China). A water droplet of 2 μL was carefully injected onto the film surface. Then, an image of the droplet was captured from which a contact angle measurement could be obtained. Five different locations on each sample were tested, and the mean was taken to determine the static contact angle [38].

## 3. Results and Discussion

**Figure 2:**
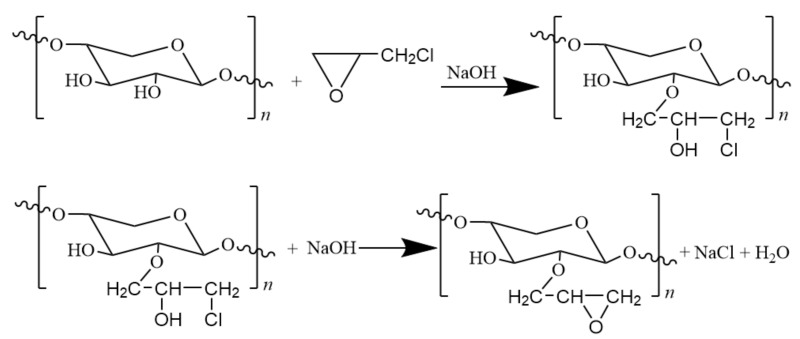
The etherification reaction of hemicellulose with epoxy chloropropane.

**Figure 3:**
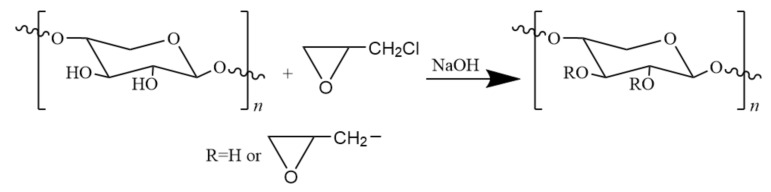
The main reaction of etherification of hemicellulose with epoxy chloropropane.

**Figure 4:**
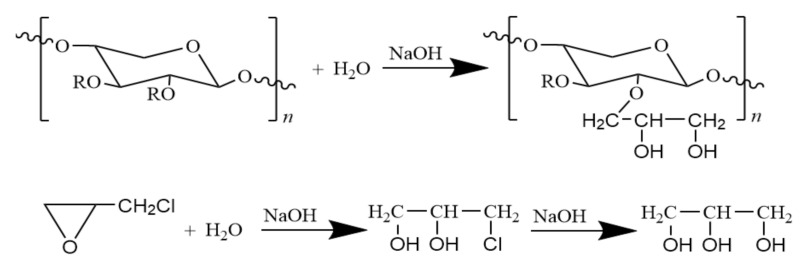
The side reactions of etherification of hemicellulose with epoxy chloropropane.

**Figure 5:**
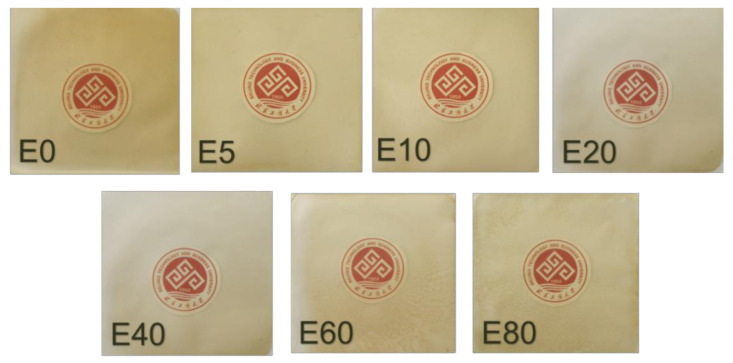
Photos of etherification modified poplar hemicellulose films.

The etherification reaction of hemicellulose, where epoxy chloropropane was used as alkylation reagent, proceeded under basic conditions and underwent a process of ring opening, condensation reaction and ring closing. The equations of these steps are as follows (Figure 2):

In this etherification approach, hydroxyl groups of hemicellulose are used as electrophiles to react with epoxy chloropropane. Due to their extremely weak acidity, they can only react under mild conditions in the presence of an alkaline catalyst. The main and side reactions of the etherification process are as follows (Figure 3 and Figure 4):

Following the etherification modification of hemicellulose, a series of hydrophobilized poplar hemicellulose films were prepared by a solution casting method, as shown in Figure 5. It can be seen from Figure 5 that the transparency of the etherified hemicellulose film, especially E20 and E40, is improved compared with the unmodified hemicellulose film (E0). The effect and mechanism of epoxy chloropropane addition on the film structure, mechanical properties, barrier properties and heat resistance were studied.

### 3.1. Structural Analysis of Etherified Hemicellulose Films

**Figure 6:**
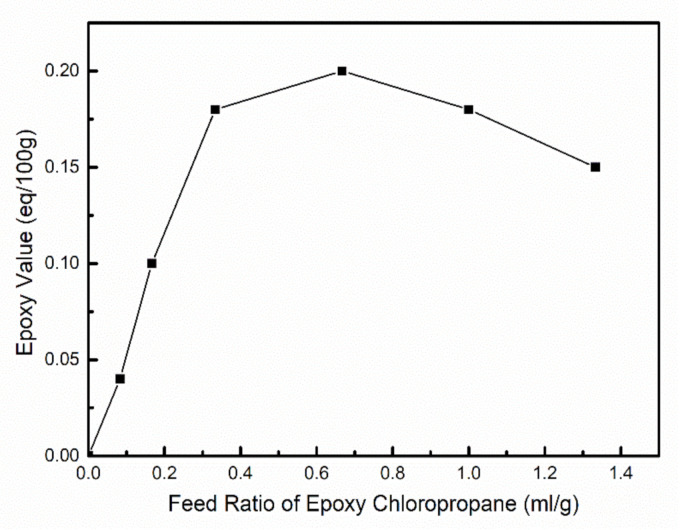
Effect of epoxy chloropropane feed ratio on the epoxy value of hemicellulose.

The content of epoxy groups in the etherified hemicellulose was measured under different proportions of epoxy chloropropane, and the influence of the ratio of epoxy chloropropane on the epoxy value of hemicellulose was analyzed, as shown in Figure 6. At a low epoxy chloropropane dosage, the epoxy value of etherified hemicellulose increased with the increase in epoxy chloropropane dosage. When the epoxy chloropropane feed ratio reached 2/3 (mL/g), the epoxy value of hemicellulose reached the maximum of 0.20 (eq/100 g). As the amount of epoxy chloropropane continued to increase, the epoxy value of hemicellulose reduced slightly.

The above phenomenon may be caused by the following two reasons: (1) epoxy chloropropane is not soluble in water, and the etherification of hemicellulose was carried out in a heterogeneous reaction system. When the amount of epoxy chloropropane is low, the low probability of two-phase contact made etherification reacting difficult, leading to a low epoxy value of hemicellulose. With the increase in epoxy chloropropane, the higher contact probability between hemicellulose and epoxy chloropropane was conducive to the etherification reaction of hemicellulose [39]. Therefore, the epoxy value of hemicellulose was improved. (2) When epoxy chloropropane was in excess, it was more likely to be alcoholyzed under alkaline conditions, and more sodium hydroxide was consumed [40]. As a result, the effective alkali concentration of the system decreased, which had an adverse effect on the epoxidation of hemicellulose. In addition, excessive epoxy chloropropane tended to form an oil film on the surface of hemicellulose, which hindered the contact between sodium hydroxide solution and hemicellulose, resulting in a slight decrease in the epoxy value of hemicellulose.

**Figure 7:**
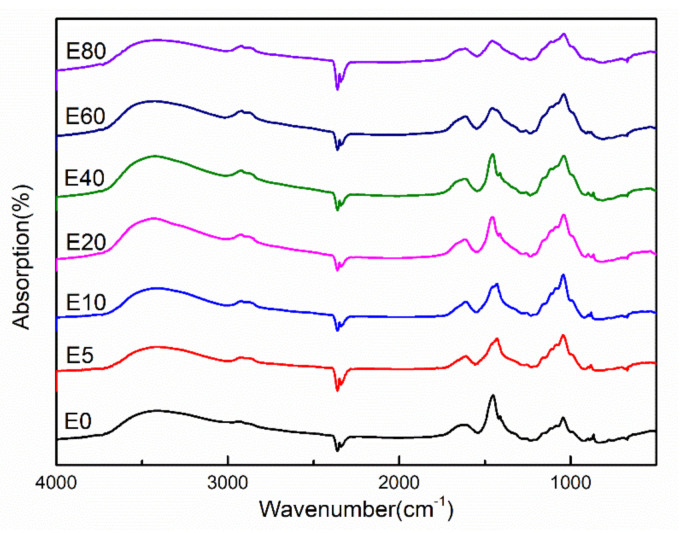
FT-IR spectra of the bank and modified hemicellulose films.

The FT-IR spectra of the etherified hemicellulose films are shown as Figure 7. The observed characteristics peaks of hemicellulose agree with those reported in literature, indicating successful extraction and modification [1,29,36]. More specifically, in the unetherified poplar hemicellulose, the C–O stretching vibration peak intensity at 1044 cm^−1^ was significantly weaker than that of the uronic acid –COO^−^ stretching vibration peak intensity at 1415 cm^−1^. However, after etherification modification, the strength of the C–O stretching vibration peak at 1044 cm^−1^ was significantly increased, which was equivalent to the strength of –COO^−^ peak at 1415 cm^−1^. This indicated that the number of C–O bonds in the modified hemicellulose increased significantly after etherification, which confirmed the occurrence of etherification modification. In addition, the C–H stretching vibration of the hemicellulose films was observed at 2927 cm^−1^. The wide peak measured at 3500 cm^−1^ was the stretching vibration peak of fatty alcohol (–OH).

### 3.2. Mechanical Properties of Modified Hemicellulose Films

**Figure 8:**
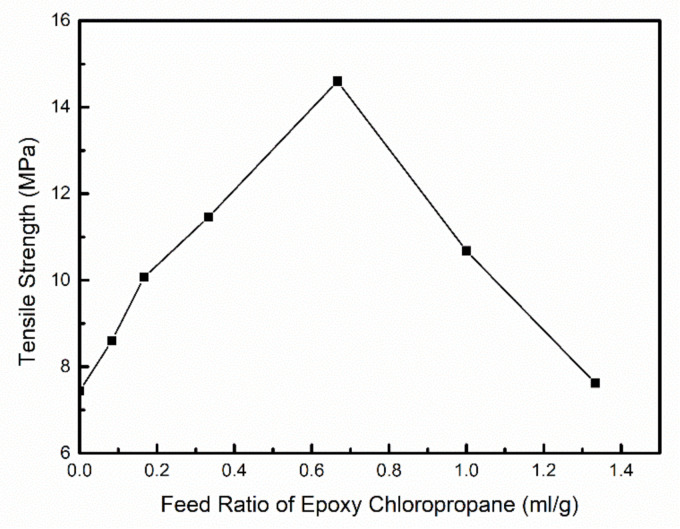
Effect of epoxy chloropropane feed ratio on the tensile strength of hemicellulose films.

The thickness, tensile strength, and elongation at break of modified hemicellulose films are summarized in Table 2 and Figure 8 for analysis of the effect of epoxy chloropropane dosage on mechanical properties. There was no obvious yield phenomenon in the tensile process of poplar hemicellulose films. Table 2 shows that the tensile strength of hemicellulose films increased first and then decreased with the increase in the amount of epoxy chloropropane, while the elongation at break of the film showed no significant changes.

Through combined analysis of Figure 6 and Figure 8, it was found that the trend of the influence of epoxy chloropropane dosage on the epoxy value of hemicellulose, was basically consistent with the trend of its influence on the tensile strength of the film. This indicates that the tensile strength of the hemicellulose films is positively correlated with the content of the epoxy groups in the hemicellulose. This may be because the epoxy groups replaced the hydroxyl groups in the process of the etherification of hemicellulose, meaning the rigidity of the hemicellulose molecular chain increased, and slip between the molecular chains was more difficult. To some extent, the tensile strength of the hemicellulose films was strengthened. When the feed ratio of epoxy chloropropane reached 2/3 (mL/g), the epoxy value of etherified hemicellulose was the highest, and the tensile strength of the film reached the peak (14.6 MPa) at the same time.

### 3.3. Thermal Stability Analysis of Modified Hemicellulose Films

**Figure 9:**
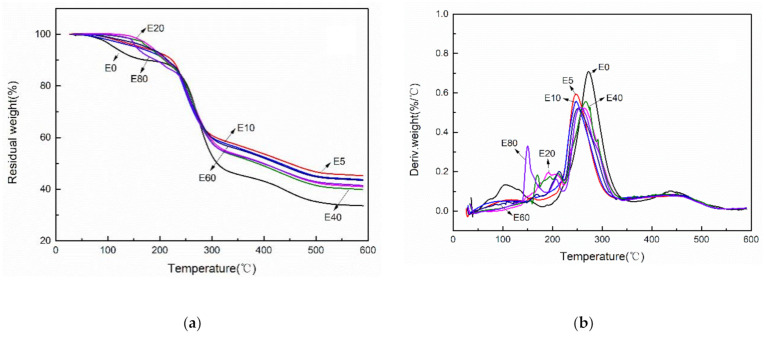
The thermal stabilities of modified hemicellulose films (**a**) TGA curves; (**b**) DTG curves.

The thermal stabilities of modified hemicellulose films were investigated by TGA. As shown in the TGA curves of modified films (Figure 9), the weight loss of all the films were observed at three stages, i.e., 60–220 °C (water evaporation), 220–350 °C (main stage of weightlessness) and 350–550 °C (carbonization process).

In the temperature range of 60–220 °C, E0 film showed the largest weight loss because most water was absorbed by unmodified film. Whilst under the same drying conditions, the weight loss of etherified films in 60–220 °C decreased significantly, proving the hydrophobic effect. In a higher temperature range of 220–350 °C, the weight loss was mainly due to the cleavage of C–O bonds and C=O bonds of polymer side chains. In an even higher temperature range, 350–550 °C, the weight loss was attributed to the cleavage of the C–C backbone of the polymer, i.e., so-called carbonization [41]. The amount of carbon residue in 600 °C of etherified hemicellulose films was higher than that of unmodified film.

The maximum weight loss temperatures (*T*_max_) of all films were observed in the range of 250–280 °C in DTG curves. The thermal decomposition rate at *T*_max_ of etherified films was significantly lower than that of unmodified film, showing that etherified modification could improve the thermal performances of poplar hemicellulose. In addition, different dosages of epoxy chloropropane influenced the thermal properties of poplar hemicellulose. When the feed ratio of epoxy chloropropane reached 1/3 (mL/g) and above, a new peak appeared in the region of 140–230 °C, which may be due to the decomposition of the introduced epoxy groups, followed by the cleavage of the polymer side chains.

### 3.4. Surface Hydrophobicity Analysis of Modified Hemicellulose Films

**Figure 10:**
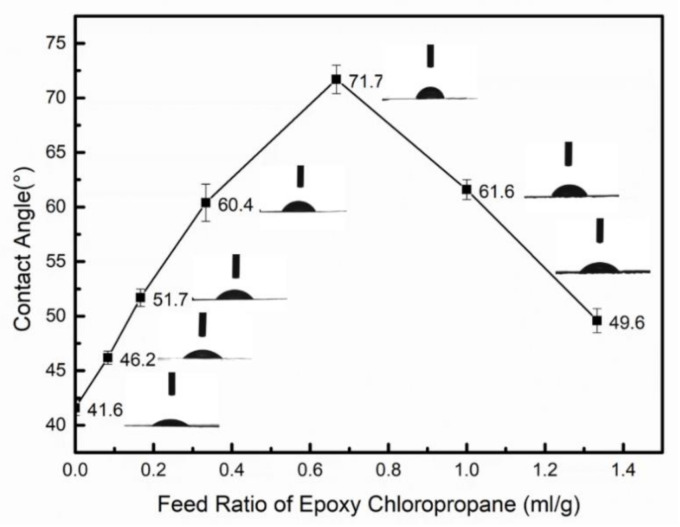
Contact angle results of modified hemicellulose films.

For evaluating the hydrophobicity of modified hemicellulose films, the contact angle of the film was measured by the sessile drop method. Five different locations on each sample were tested and the mean was taken as the static contact angle. The contact angles were measured to be 41.6°, 46.2°, 51.7°, 60.4°, 71.7°, 61.6° and 49.6° for E0, E5, E10, E20, E40, E60 and E80, respectively (Figure 10).

When comparing Figure 6 and Figure 10, it was found that the trend of the influence of epoxy chloropropane dosage on the epoxy value of hemicellulose was basically consistent with that of its influence on the contact angle of the film. This is mainly because as more hydroxyl groups on the hemicellulose molecular chain are replaced with epoxy groups, the polarity decreases as does the surface energy. Due to the repulsive force, the non-polar surface will produce a larger angle [42], so the contact angle increases. Among them, the contact angle of E40 film was the highest (71.7°) with the feed ratio of epoxy chloropropane at 2/3 (mL/g), which is an increase of 30.1° from the original film. The increased contact angle with the addition of epoxy group indicates a significant reduction in the hydrophilicity of hemicellulose films.

### 3.5. Oxygen Resistance Performance of Modified Hemicellulose Films

Good oxygen resistance performance is essential for food-packaging materials to avoid the oxidation and deterioration of food. The oxygen permeability (OP) values of the etherified poplar hemicellulose films and some typical packaging materials are summarized in Table 3.

The OP values of hemicellulose-based films were far lower than that of LDPE (low density polyethylene), which represented typical petroleum-based packaging material. This reflected the superiority of hemicellulose as a food packaging material. With the increase in epoxy group content, the oxygen transmission coefficient of modified poplar hemicellulose film decreased significantly. When the feed ratio of epoxy chloropropane reached 2/3 (mL/g), the OP value of modified poplar hemicellulose film was 1.9 (cm^3^·µm)/(m^2^·d·kPa), lower than other biomass films mentioned in Table 3. This may be because the introduction of epoxy groups enhanced the adhesion within the hemicellulose film and reduced the intermolecular gaps, thus improving the performance of the film as an oxygen barrier.

## 4. Conclusions

The etherified hemicellulose was directly extracted and modified from poplar powder by a one-step method to avoid the destruction of hemicellulose structure by secondary alkali-hydrolysis. A series of modified hemicellulose films were prepared by a solvent casting method, and the barrier properties and thermal stability of the modified films were improved significantly, showing great potential for application in the field of food-packaging materials.

Among them, when the ratio of the volume of epoxy chloropropane (mL) to the weight of delignified wood powder (g) was 2/3, the epoxy value of modified hemicelluloses was the largest. As a result, the film had the highest tensile strength of 14.6 MPa, the maximum surface contact angle 71.7° and the lowest oxygen transmission coefficient of 1.9 (cm^3^·µm)/(m^2^·d·kPa). The etherified modified hemicellulose film had barrier properties comparable to traditional petroleum-based film, but there is still a gap in mechanical properties [32].

In this work, the feasibility of etherification within hemicellulose and epoxy chloropropane was confirmed and the reaction mechanism was explained. Subsequently, the epoxy chloropropane could play the role of intermediate, grafting other polymers on the hemicellulose chains by the ring-opening reaction of the epoxy group. The more opportunities given to hemicellulose could expand its prospective applications in the area of food packaging.

## Figures and Tables

**Table 1:** Designation of etherified hemicellulose films.

Sample	Dosage of Epoxy Chloropropane (mL)	Feed Ratio of Epoxy Chloropropane (mL/g)	Mass of Hemicellulose (g)	Mass of PVA (g)	Mass of Sorbitol (g)
E0	0	0	0.90	0.30	0.30
E5	5	1/12	0.90	0.30	0.30
E10	10	1/6	0.90	0.30	0.30
E20	20	1/3	0.90	0.30	0.30
E40	40	2/3	0.90	0.30	0.30
E60	60	1/1	0.90	0.30	0.30
E80	80	4/3	0.90	0.30	0.30

**Table 2:** Tensile test results of the modified hemicellulose films.

Sample	Tensile Strength (MPa)	Elongation at Break (%)	Sample	Tensile Strength (MPa)	Elongation at Break (%)
E0	7.44 ± 0.44	4.36 ± 0.06	E40	14.60 ± 1.33	2.53 ± 0.22
E5	8.60 ± 0.59	5.65 ± 1.19	E60	10.68 ± 0.37	5.05 ± 0.65
E10	10.07 ± 0.83	5.53 ± 0.84	E80	7.62 ± 0.66	6.12 ± 0.52
E20	11.46 ± 1.90	5.48 ± 1.32			

**Table 3:** Comparison of oxygen permeability of the packaging films in literatures and this work.

Sample	Oxygen Permeability [(cm^3^·µm)/(m^2^·d·kPa)]	Reference Citation
E0	1053 ^1^	-
E5	204 ^1^	-
E10	26.5 ^1^	-
E20	4.3 ^1^	-
E40	1.9 ^1^	-
E60	5.2 ^1^	-
E80	18.6 ^1^	-
Spruce galactoglucomannan	6.8	[43]
Oat spelt arabinoxylan with 40% sorbitol	4.7	[44]
*O*-acetyl-galactoglucomannan with nanofibrillated cellulose	3.2	[45]
Hydroxypropylated birch xylan	4.7–24	[46]
Quaternized hemicelluloses with chitosan and montmorillonite	10.95–16.37	[1]
Low-density polyethylene (LDPE)	7900	[47]
Poly(lactic acid) (PLA)	160	[47]
Poly(hydroxyalkanoate) (PHA)	150	[47]

^1^ Test conditions: 23 °C, 50% RH.
